# Validation of an accelerometer system for activity monitoring in children with functional disabilities

**DOI:** 10.1007/s00431-025-06679-4

**Published:** 2026-01-15

**Authors:** Anna Stage, Emil Buch Fromberg, Peter Elsborg, Mette Røn Kristensen, Silje Mikkelsen, Mads Bølling, Mette Aadahl, Michelle Stahlhut

**Affiliations:** 1 Center for Clinical Research and Prevention, Bispebjerg and Frederiksberg Hospital, Copenhagen, Denmark; 2 Department of Nutrition, Exercise, and Sports, University of Copenhagen, Copenhagen, Denmark; 3 Interdisciplinary Competence Center - Health Care Services, Allerød Municipality, Allerød, Denmark; 4 Center for Child and Youth Services, Educational Psychological Counseling, Ballerup Municipality, Ballerup, Denmark; 5 Research Centre for Didactics and Pedagogy, Program On Outdoor Pedagogy, VIA University College, Aarhus, Denmark; 6 Department of Clinical Medicine, Faculty of Health and Medical Sciences, University of Copenhagen, Copenhagen, Denmark

**Keywords:** Validation, Accelerometry, Physical activity, Sedentary behavior, Functional disabilities, Children

## Abstract

To assess the validity of the SENS motion® system (SENS) for measuring postures and movements in school-aged children with functional disabilities, using direct video observation as the criterion method. In this cross-sectional-study, 29 children (51.7% male, mean age 10.8 ± 2.9 years) from two special schools in Denmark participated. Each child wore a SENS device on the thigh while completing a standardized protocol of six categories: lying/sitting, standing, walking, running, cycling, and step count. All activities were video recorded. Video data were coded in 5-s epochs and aligned with SENS output. Agreement was assessed by comparing the observed time spent in each activity with the corresponding time estimated by SENS, expressed as mean values, standard deviations, mean differences, and percentage agreement. SENS showed excellent agreement for walking (93.2%) and lying/sitting (96.2%), good agreement for running (89.2%), and moderate agreement for standing (74.1%). Cycling was poorly detected, with only 6.4% agreement, as it was frequently misclassified as walking (50.8%) or lying/sitting (42.7%). Step counts were slightly overestimated by SENS (mean difference 7.2 steps). Overall, SENS tended to underestimate activity duration compared with the observation. *Conclusion*: SENS demonstrated excellent to good validity for detecting lying/sitting, walking, and acceptable validity for running in children with functional disabilities, but moderate validity for standing and limited validity for cycling. These findings indicate that SENS may be useful for monitoring several common postures and activities in this population, though further algorithm refinement and broader validation are needed, particularly for cycling and postural transitions.

**Table:** 

What is Known: • Children with disabilities engage in less dynamic postures and more lying/sitting time than their peers without disabilities. • Accelerometer systems are typically validated in healthy populations, limiting accuracy in children with disabilities. What is New: • SENS shows excellent to good validity for lying/sitting, walking, and running in children with functional disabilities. • SENS performs poorly for cycling and moderately for standing, highlighting the need for algorithm refinement.

What is Known:

• Children with disabilities engage in less dynamic postures and more lying/sitting time than their peers without disabilities.

• Accelerometer systems are typically validated in healthy populations, limiting accuracy in children with disabilities.

What is New:

• SENS shows excellent to good validity for lying/sitting, walking, and running in children with functional disabilities.

• SENS performs poorly for cycling and moderately for standing, highlighting the need for algorithm refinement.

## Introduction

Physical activity (PA) and sedentary behavior (SB) are key determinants of health and wellbeing across the lifespan [1]. In childhood, regular PA contributes to physical, cognitive, and social development, whereas prolonged SB is associated with adverse health outcomes including obesity, poor cardiometabolic profiles, and reduced psychosocial wellbeing [1–3]. Children with functional disabilities are particularly vulnerable, as evidence consistently demonstrates lower levels of PA and higher levels of SB compared to their typically developing peers [4–6]. Contributing factors include mobility limitations, environmental barriers, and restricted access to inclusive programs [4, 7, 8]. As a result, this population faces a disproportionate risk of insufficient PA levels and an increased likelihood of chronic health conditions [9].

Accurate and reliable measurement of postures and movements are essential for monitoring movement patterns, evaluating interventions, and informing health promotion strategies. Accelerometry has emerged as the preferred method in both clinical and population-based research, providing objective, time-stamped information on movement and posture [10–12]. However, most accelerometer devices and classification algorithms have been developed and validated in populations with typical development. This poses a challenge when assessing children with functional disabilities, who often display irregular, low-intensity, or atypical movement patterns that can compromise algorithm performance and result in misclassification [13–16]. Systematic reviews have highlighted the lack of validated accelerometer protocols specifically tailored to children with physical or intellectual disabilities [7, 10, 12].

Many types of accelerometers exist, and it is important to evaluate validity, characteristics, and cost to choose the most suitable one [17]. The SENS motion® system (SENS) is a recently developed thigh-worn accelerometer that offers practical advantages such as extended battery life, continuous data transfer, and minimal user burden through the use of a custom-made adhesive patch [18]. Importantly, SENS employs an automated non-code classification algorithm to distinguish between fundamental activity types and postures. Initial validation studies in adults and children without disabilities have shown promising results. For example, Lendt et al. (2024) reported substantial agreement between SENS and video observation in adults without disabilities in both laboratory and free-living conditions, with balanced accuracy ranging from 0.81 for cycling to 0.99 for running [19]. Similarly, a recent study in children and adolescents without disabilities demonstrated strong agreement between SENS and observation for walking, sitting time, and lying time, with intraclass correlation coefficients ranging from moderate to excellent (0.50–0.94) and low standard error of measurement for most outcomes [20]. However, weaker agreement was observed for fast walking, sitting, and step counts, suggesting that the system may have limitations for detection of certain postures and movements. In adult clinical populations without disabilities, Bartholdy et al. demonstrated very high agreement for sitting but low accuracy for walking detection in patients with knee osteoarthritis [21], while Pedersen et al. reported a high percentage agreement for walking time and steps in hospitalized patients with slow gait speeds [22]. Consequently, establishing the validity of SENS in children with functional disabilities is essential to ensure accurate classification of postures and movements to strengthen the evidence base for intervention studies targeting populations with disabilities.

Therefore, the aim of this study was to assess the validity of the SENS motion® system in school-aged children with functional disabilities by comparing its output to direct video observation.

## Materials and methods

### Study design and participants

This cross-sectional study aimed to assess the concurrent validity of SENS using direct video observation as the criterion measure. Participants were recruited from two special schools in the Capital Region of Denmark using convenience sampling. To be included, the children had to be between 6 and 18 years, have a confirmed diagnosis resulting in functional disabilities, and have the ability to stand and/or walk independently or with assistance. Children were not eligible if they had undergone orthopedic surgery in the lower extremities within the last six months or had undergone spinal fusion within the last 12 months.

The parents of participating children received an information letter about the study including a consent form a month prior to the test day.

**Fig. 1:**
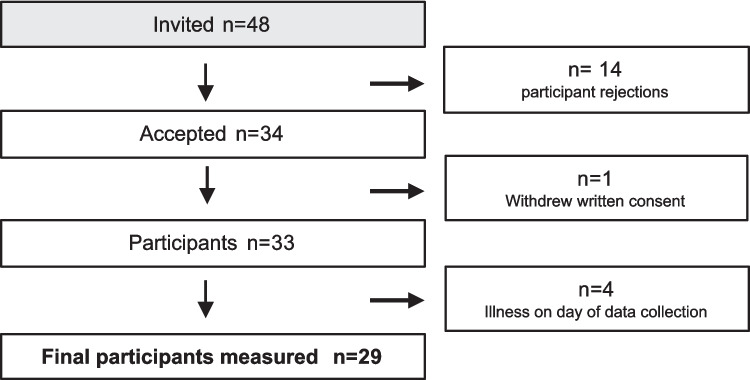
Flowchart of the number of invited and participating participants

A total of 48 students were invited to participate, of whom 34 had a parent-signed consent form. Four participants were absent due to illness on the day of testing, and the parents of one participant withdrew their consent. Therefore, the final number of participants included was 29 (Fig. 1).

Of the included participants, 51.7% were male, and the age ranged from 6.4 to 16.9 years (mean 10.8 years, SD 2.9 years). Most participants were independently ambulant (65.5%), while 17.2% required assistance and 17.2% were non-ambulant. The most common diagnostic categories were neurological disorders (48.3%) and genetic syndromes or mutations (37.9%), (see Table 1).

**Table 1:** Characteristics of included participant *n* = 29

	Mean (SD)	Number (%)
Age (years)	10.8 (2.9)	
Sex		
Male Female		15 (51.7) 14 (48.3)
Ambulation level		
Independent Assisted^a^ Non-ambulant		19 (65.5) 5 (17.25) 5 (17.25)
Diagnoses		
Neurological disorders (e.g., cerebral palsy, acquired brain injury) Genetic syndromes and mutations (e.g., cornelia de Lange) Motor disorders and coordination difficulties (e.g., dyspraxia) Developmental and intellectual disabilities (unspecified)		14 (48.3) 11 (37.9) 2 (6.9) 2 (6.9)

^a^ Participants who were categorized as walking with assistance used walking aids (*n* = 2 anterior walkers, *n* = 2 posterior walkers, *n* = 1 walker with seat)

### Data collection

Three of the authors (MRK, SM, MS) were responsible for participant inclusion, data collection, and assessment. Data collection took place over six days between September and November 2023. Participants’ age, sex, and diagnosis were recorded. Participating children were asked to complete a structured activity protocol during a school day (see Table 2). Each participant was equipped with a SENS accelerometer, positioned on the right lateral thigh approximately 10 cm above the lateral epicondyle using a customized adhesive plaster. Two participants had it placed on the left thigh as they were hemiplegic with an affected right lower limb.

**Table 2:** Activities performed during the structured protocol at the school

Activity	Description	Duration (s)
Sitting	Sitting on a chair close to a table (doing a puzzle or drawing allowed during the last 90 s)	180
Lying	Lying supine on couch	120
Standing	Standing independently or using standing frame	120
Walking	Walking back and forth in gym at own preferred walking speed. The walking activity is used to count number of steps	120
Running	Running back and forth in gym at own preferred running speed	60
Biking	Cycling on a stationary bike in gym	120

Following the structured activity protocol, participants completed six consecutive activities (Table 2). The trial was conducted in the gymnasium of each school, with participants accompanied by their regular physical or occupational therapist. All activities were performed with accelerometers attached to the participants thigh and the activities were simultaneity recorded using a smartphone camera, with video observation selected as the optimal method for comparison with SENS to assess concurrent validity [23].

#### Activity protocol

The activities were selected to evaluate the sensor’s ability to classify postures and movements (Table 2). The activities included in this study are relevant, fundamental, and important, aligning with those used in similar validity studies [20, 24, 25]. Additionally, the selection was based on the predefined activity categories within the SENS algorithm [18].

One researcher (MS or MRK) video recorded the child while he/she performed the structured protocol, while another researcher (SM or MS) and the physical or occupational therapist gave the child instructions and encouragement during the activities. Table 2 shows the activities performed during the structured protocol. Children who were non-ambulant only performed the sitting, lying, and standing activities. If required, the child’s favorite music or cartoon was played during the activities to enhance cooperativeness and motivation. As some of the participants were not able to perform all the activities due to mobility restrictions, the number of participants performing the activities varied.

#### Video observation

The video recordings were performed with a video camera on an Apple Iphone SE (2020) Black 64 GB, with a sampling frequency of 60 Hz. One video sequence was recorded for each activity, and the recording was paused between activities.

### SENS accelerometer

SENS is a small waterproof sensor (45 × 21 × 5 mm, 6 g), with a triaxial accelerometer. It has a battery lifetime of 15 weeks, and a data storage capacity to save recorded data continuously for a period of 14 days, if the sensor is out of range of a smartphone. When the sensor is within 15 m of a smartphone, it connects wirelessly, and data are transmitted to the application every 10 min. The raw accelerometer data are automatically uploaded to a secure web server via the smartphone’s wifi connection. The raw acceleration is calculated as the average magnitude of movement based on high-pass filtered three-axis accelerometer measurements at a 12.5 Hz sampling frequency.

The SENS algorithm classifies activity based on the raw triaxial acceleration from a single thigh-worn sensor. Every 5 s, it calculates the thigh inclination (low-frequency part of the signal) and the movement intensity (high-frequency part of the signal). Postures (lying/sitting vs. upright) are identified primarily from the thigh inclination relative to gravity (≈ < 30° = lying/sitting, > 40° = upright). Within upright periods, dynamic acceleration patterns are then used to separate activity types: low intensity with no periodic signal = standing, periodic patterns with increasing movement intensity = walking and running (with separate cut-offs for intermittent steps, continuous walking, moderate and high intensity movement). Cycling is recognized when the thigh exhibits smooth periodic rotation without step-related impact peaks. In short, the classifier first uses orientation to identify posture, and then uses the frequency domain characteristics and intensity of the dynamic signal to differentiate walking, running, and cycling.

The predefined postures and movements are: lying sitting rest, lying sitting movement, upright stand, upright sporadic walk, upright walk, upright moderate, upright run, cycling, and steps, steps2, steps3 [18]. Please see Table 3 for detailed information on the SENS activity categories.

**Table 3:** Definition and interpretation of SENS activity categories [18]

SENS activity categories	Definition	Interpretation
Lying_sitting_rest	Sensor in horizontal position (± 45°) with intensity count < 2	Participant is sitting or lying still
Lying_sitting_movement	Sensor in horizontal position (± 45°) with intensity count > 2	Participant is sitting or lying with movement. Light cycling may occasionally appear here
Upright_stand	Sensor in vertical position (± 45°); intensity below ~ 0.1 G threshold	Participant is standing still with minimal movement
Upright_sporadic_walk	Upright position; movement above threshold but below moderate intensity; non-repetitive	Short or irregular upright movements (e.g., a few steps or slow, brief cycling)
Upright_walk	Repetitive pattern; intensity above threshold but below moderate level; frequency > 0.2 Hz; asymmetric leg movement	Continuous walking for 5–10 s intervals. Shorter sequences (3–9 s) are classified as sporadic walking
Upright_moderate	Activity classified as walking over 3–9 s with higher intensity than normal walking but below high-intensity threshold	Moderate-intensity upright activity (e.g., brisk walking)
Upright_run	Continuous walking-type activity above moderate-intensity range for ≥ 3–9 s (not cycling)	Running or vigorous leg-intensive activity
Cycling	Repetitive symmetric leg movement, frequency > 0.2 Hz, intensity above threshold	Continuous cycling for ≥ 1–2 min. Shorter cycling bouts may appear as walking or sporadic walking
Steps	Steps detected during continuous walking or training (based on frequency-domain analysis over 5 s)	Continuous walking steps
Steps1	Steps during sporadic walking (no consistent frequency pattern); estimated as 2 steps per 5 s interval	Irregular or short walking bouts
Steps2	Steps during low-intensity walking with a detectable continuous frequency pattern (5 s window)	Low-intensity, continuous walking steps

### Data processing

SENS offers a non-code system with a predefined algorithm. A non-code system means that once data are uploaded to the cloud platform, users can automatically process and retrieve the results through the predefined algorithm, without the need for manual coding or programming. Data was sampled every fifth second and the predefined categories were combined as follows: (1) “Lying/sitting” in which “lying sitting rest” and “lying sitting movement” were combined; (2) “Standing” which were upright stand; (3) “Walking” which were “upright sporadic walk”, “upright walk” and “upright moderate” combined; (4) “Running” which were upright run; (5) “Cycling” which were cycling; and (6)”Step count” was “step1”, “step2″ and “step3″ combined.

Two of the authors (MRK and EBF), both physiotherapists experienced in pediatric and clinical movement analysis, reviewed the videos and classified each five-second segment according to the predefined categories (lying/sitting, standing, walking, running or cycling). If a participant performed more than one category within a five-second segment, the activity with the longest duration was selected. Additionally, steps were counted during the walking activity. In cases of uncertainty, the coders discussed the classification with the project leader (MS), also a pediatric physiotherapist, until consensus was reached. This consensus-based approach was applied to enhance the consistency and accuracy of the video coding. Data were obtained only during the period in which the protocol was performed. Specifically, once the walking segment of the protocol began, the subsequent 120 s were collected and categorized, regardless of whether participants continued the activity beyond this period or deviated from it. If a participant performed an activity other than the designated one within this timeframe, the corresponding data were still included and categorized according to the activity actually performed. SENS data were synchronized by matching the time recorded to the timestamps on the video recordings. This was used as the criterion measure for concurrent validation.

### Data analysis

Descriptive statistics of total seconds, mean (SD), mean difference (SD) and percentage agreement of mean time for SENS and observation were calculated. This was visualized in Table 4, with time for video observation and SENS only, reflecting the actual time the participant did the predefined activity.

**Table 4:** Performance of the protocol for the observation for SENS categorization and the comparison of SENS and video observation

		Per protocol	Video observation		SENS		Comparison	
Activity categories	*n*	Total	Total	Mean (SD)	Total	Mean (SD)	Mean difference (SD)	Agreement (%)
Lying/sitting (seconds)	23	6900	6900	300 (0)	6660	289.6 (34.6)	10.4 (34.6)	96.5
Standing (seconds)	28	3360	3300	117.9 (8.3)	2535	90.5 (37.7)	27.3 (34.7)	76.8
Walking (seconds)	22	2640	2500	113.6 (9.8)	2480	112.7 (11.6)	0.91 (10.5)	99.2
Running (seconds)	18	1080	915	50.8 (17.3)	880	48.9 (18.3)	1.9 (21.4)	96.2
Cycling (seconds)	16	1920	1795	111.9 (13.8)	115	7.2 (28.8)	104.7 (29.9)	6.4
Steps (counts)*	22		3872	176 (46.9)	4052	184.2 (56.2)	−8.2 (22.8)	95,5*

*Steps refer to the count of steps during walking

To evaluate the agreement between video observed postures and movements and those classified by the accelerometer, a confusion matrix was constructed for the total protocolled period no matter prescribed activity. The confusion matrix tabulates the percentage of each video observed postures and movements measured in 5-s intervals against the predicted label from the accelerometer measured in seconds, allowing a direct comparison of classification performance across activity types. If a participant deviated from the predefined activity, both the video recordings and SENS data were classified according to the activity actually performed (e.g., standing instead of sitting). Consequently, the total number of measured seconds differs between the video observations and the SENS data, as shown when comparing Table 4 and the confusion matrix.

An agreement of > 90% was considered excellent, 80% to 90% good, 70% to 80% moderate and less than 70% poor [26]. All analyses were conducted in R Studio 4.4.2.

## Results

When comparing total seconds and mean time spent in each of postures and movements from both the video observation and from SENS, the best percentage agreement between the two methods was seen in the walking category with 99.2%, then the running category with 96.2%, and lying/sitting with 91.3%. The highest mean difference in time was found for standing with 27.3 seconds (76.8%) and cycling with 104.7 seconds (6.4%), respectively. Overall, SENS recorded less time per designated activity than observation, except for step count, which was overestimated by a mean of 8.2 steps.

The confusion matrix (Table 5) shows percentage of agreement between postures and movements from SENS and video observation with the diagnoal being agreement between the two methods. The highest agreement, as considered exellent was seen for lying/sitting with 96.2% and walking with 93.2%. Running was considered good with 89.2% and moderate for standing with 74.1%. The confusion matrix shows that some of the time standing was misclassified into walking (24.8%), and that running was incorrecly estimated as walking 10.8% of the time. Cycling showed a poor agreement of 6.4%, where 50.8% was misclassifed as walking and 42.7% as lying/sitting by SENS.

**Table 5:**
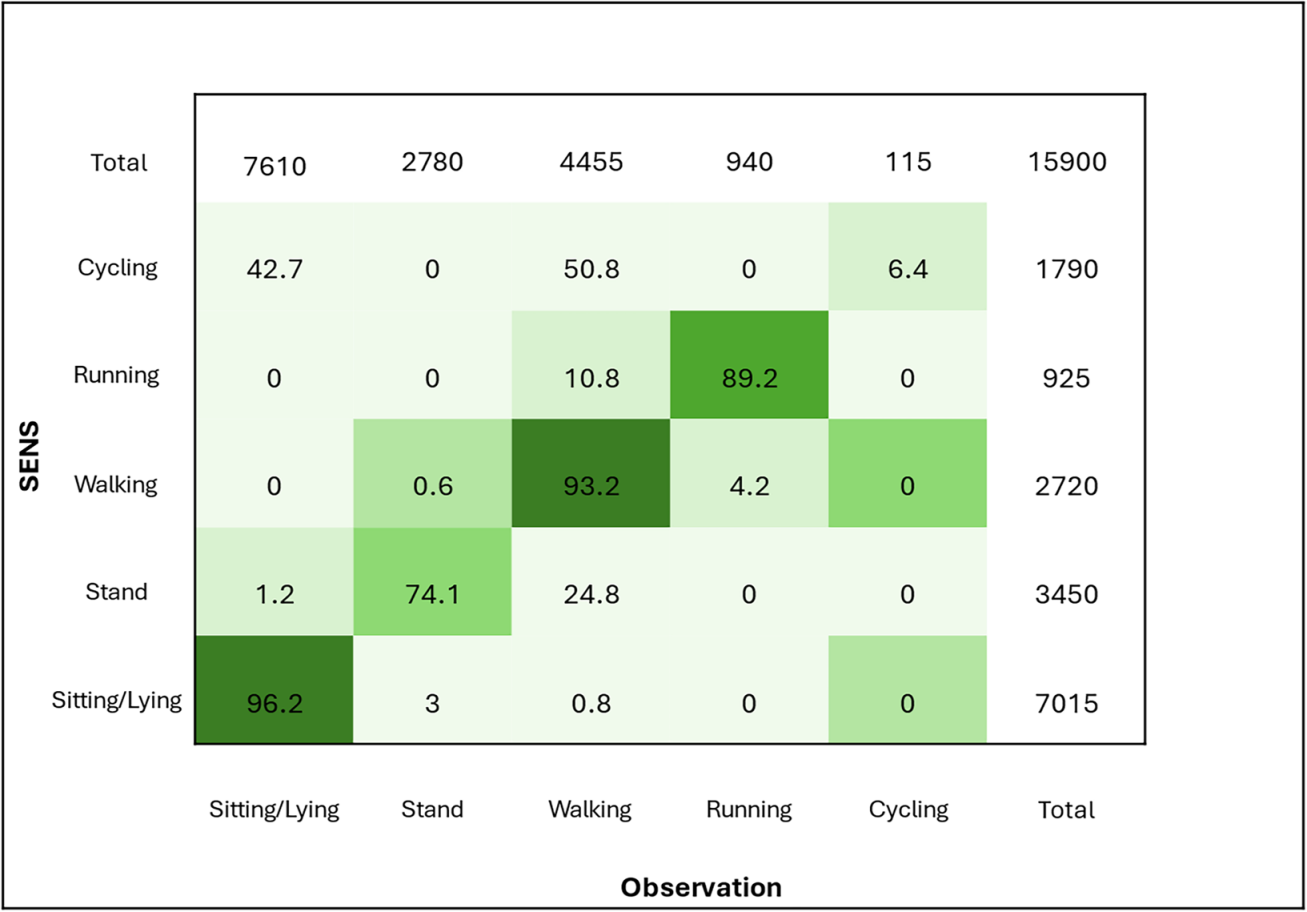
Confusion matrix of SENS accelerometer categorization percentage to observation categorization

Row represents the reference video observation, and columns represents the estimated activity types from SENS. Values represent row precentage

## Discussion

This study examined the concurrent validity of the SENS accelerometer compared with direct observation in school-aged children with functional disabilities. Participants completed a structured activity protocol consisting of six physical activities while wearing the SENS device mounted laterally on the thigh. Overall, the system demonstrated excellent to moderate validity for lying/sitting, standing, walking, and running, with classification accuracies ranging from 74.1% to 96.2%. However, for cycling the performance was poor, with an accuracy of only 6.4%.

The strongest results were observed for lying/sitting (96.2%) and walking (93.2%), indicating that the SENS system can validly capture these key postures and movements in children with functional disabilities. Running was also well detected (89.2%), while standing showed only moderate agreement (74.1%). Misclassification in standing may be due to subtle weight shifts or atypical postural control in this population, which can be difficult for accelerometer algorithms to distinguish from light ambulatory activity [27, 28]. In this population, some participants exhibited increased hip spasticity, which may have made it challenging for the device to accurately detect upright postures. As a result, these positions could have been misclassified as sitting due to the flexed hip position. Cycling presented a particular challenge, where the SENS device frequently misclassified cycling as walking (50.8%) or lying/sitting (42.7%). This limitation is consistent with previous reports that thigh-mounted accelerometers struggle to accurately detect cycling movements, likely due to limited vertical displacement during pedaling [29]. Furthermore, in some cases, the stationary bicycle appeared to be adjusted too low, potentially restricting hip extension among participants with contractures. Importantly, the SENS system performed well in detecting step counts, counteracting earlier concerns that accelerometers may underestimate steps in individuals with atypical gait patterns, such as those with Rett syndrome [30].

These findings align with previous studies as Engels et al. (2025) similarly found strong performance for lying/sitting and walking in children with and without developmental disabilities, while highlighting challenges in detecting cycling and standing [31]. Likewise, Lankhorst et al. (2019) reported that commercial accelerometers such as Activ8 could reliably distinguish static from dynamic activities but showed limitations for more complex movements and cycling in children with and without motor disabilities [29]. More broadly, systematic reviews have underscored the scarcity of accelerometer validation studies in children with disabilities [7, 10], emphasizing the novelty and contribution of the present work. By extending validation evidence to children with functional disabilities, our study addresses a critical methodological gap and provides empirical support for the use of SENS in this population.

## Implications for practice and future research

From a practical perspective, our results suggest that SENS has potential as a valid tool for monitoring postures and movements in children with disabilities, particularly in school settings where feasibility and low participant burden are important. However, further algorithm development is needed to improve classification of cycling and more nuanced postural transitions, such as sit-to-stand, which are relevant in daily life [32]. Future research should also focus on larger and more diverse samples across different ages, disability types, ambulation levels, and settings to establish the generalizability of our findings. The potential of multi-sensor or hybrid systems for activity classification in populations with atypical movement patterns also warrants further exploration [29, 31].

## Strengths and limitations

Several strengths support the validity of our findings. The study was conducted in a school setting, using activities that reflect daily life for children with functional disabilities, thereby enhancing ecological validity. Direct observation was employed as the reference criterion, which is widely considered the gold standard in activity validation studies [33]. Furthermore, the use of confusion matrix analyses allowed for transparency and detailed insight into classification accuracy across specific activities, offering a nuanced understanding of the system’s strengths and weaknesses.

Nevertheless, some limitations should be acknowledged. The poor performance for cycling is likely influenced by the limited adjustment options of the stationary bicycle and the lateral thigh placement of the sensor, which does not optimally capture stationary pedaling movements [29]. In addition, the classification algorithm applied was not specifically adapted to atypical movement patterns common in children with disabilities, such as crouch gait or toe-walking, which may have reduced accuracy [31]. Our sample included relatively few non-ambulant children, limiting the extent to which findings can be generalized to children who primarily sit or lie down. The use of convenience sampling in a school-based setting may also restrict representativeness. Another consideration is the inclusion of children with different diagnoses. This approach reflects the heterogeneity seen in school and rehabilitation settings, enhancing the ecological validity and practical relevance of the study. Although a more homogeneous sample might reduce variability, even within diagnostic groups like cerebral palsy, motor function varies widely with GMFCS level and individual characteristics [34]. Thus, heterogeneity in movement is inherent to pediatric disability populations. Including participants with varied diagnoses provides a realistic representation of this diversity but may also have contributed to variability in classification accuracy. Furthermore, only one video sequence was recorded per activity, with the recording paused between activities. This approach reduced within-activity variation in the observational data, which may have limited the robustness of the validation. Moreover, transitions between activities (e.g., lying to standing) were not systematically recorded and therefore not included in the analysis. This omission may have limited the assessment of the system’s performance during brief or transitional movements, which are frequent in everyday life. Finally, the short duration of the standardized activity protocol does not fully reflect habitual daily activity patterns, which often include spontaneous and variable movements.

## Conclusion

The SENS motion® system demonstrated good to excellent validity for detecting lying/sitting and walking, and acceptable validity for running, while also showing moderate validity for standing and poor validity for cycling in children with functional disabilities. Overall, SENS appears to be a promising tool for monitoring certain postures and activities in this population. However, the system’s limitations, especially in detecting cycling and postural transitions, highlight the need for further refinement of algorithms and validation in more diverse contexts.

## Data Availability

Data sets generated during the current study are available from the corresponding author on reasonable request.

## Abbreviations

PA: Physical activity
SENS: SENS motion® system
*SB*: Sedentary behavior
